# PREDICTORS OF CHANGES IN WELL-BEING AMONG THE INTEGRATED MEMORY CARE CLINIC CLIENTS

**DOI:** 10.1093/geroni/igz038.425

**Published:** 2019-11-08

**Authors:** Mariya A Kovaleva, Melinda Higgins, Bonnie M Jennings, Mi-Kyung Song, Carolyn Clevenger, Patricia C Griffiths, Ken Hepburn

**Affiliations:** 1 Vanderbilt University School of Nursing, Nashville, Tennessee, United States; 2 Emory university, Atlanta, Georgia, United States; 3 Nell Hodgson Woodruff School of Nursing, Emory University, Atlanta, Georgia, United States; 4 Emory University School of Medicine, Atlanta, Georgia, United States

## Abstract

The Integrated Memory Care Clinic (IMCC) at Emory Healthcare is a patient-centered medical home led by advanced practice registered nurses who seamlessly provide dementia care and primary care. This analysis explored predictors of significant changes in clients’ well-being and symptoms in clients’ first-year experience at the IMCC (N=42 caregivers, three assessments over nine months). The significant changes were decreases in caregivers’ distress regarding PLWDs’ delusions (Delusions-Distress) and PLWDs’ anxiety (Anxiety-Distress), and in PLWDs’ severity of delusions, depression, and total symptom severity. Mixed linear models were used to determine significant predictors among baseline sociodemographic characteristics that correlated significantly with outcomes that changed significantly over time. Caregivers not employed outside home had lower baseline Delusions-Distress (p=0.006) and slower decline in Delusions-Distress (p=0.015). The longer PLWD needed care, the lower baseline Delusions-Distress caregivers reported (p=0.023). Caregivers not living with their PLWD reported higher baseline Anxiety-Distress (p=0.016). Caregivers not employed outside home reported lower baseline Delusions-Severity for their PLWD (p=0.006). Caregivers not employed outside home reported PLWDs’ lower baseline depression severity (p=0.026). Older caregivers reported PLWDs’ lower baseline total symptom severity (p=0.002). Increase in caregiver’s age was associated with PLWDs’ higher total symptom severity (p=0.049). For PLWD with male caregivers, total baseline symptom severity was lower compared to PLWD with female caregivers (p=0.01). These findings highlight that PLWDs’ illness duration and caregivers’ employment status, living arrangement, age, and gender may determine their perception of their PLWDs’ symptoms. Clinicians may individualize caregiver education with the knowledge of such predictors.

